# Organizational Psychological Capital in Family Firms: the Role of Family Firm Heterogeneity

**DOI:** 10.1007/s41471-021-00124-6

**Published:** 2021-11-29

**Authors:** Denise Fischer-Kreer, Andrea Greven, Isabel Catherine Eichwald, David Bendig, Malte Brettel

**Affiliations:** 1 Innovation and Entrepreneurship Group (WIN), TIME Research Area, RWTH Aachen University, Kackertstr. 7, 52072 Aachen, Germany; 2 University of Münster, Geiststraße 24, 48151 Münster, Germany

**Keywords:** Organizational psychological capital, Positive psychology, Psychological resources, Family firm, Top management team, Board tenure, M14

## Abstract

Organizational psychological capital—comprising hope, confidence, resilience, and optimism—is a vital resource for family firms in times of stress. Surprisingly, whether and how family firm idiosyncrasies impact organizational psychological capital remains unclear. Considering the theoretical paradigm of socio-emotional wealth, we investigate two important family firm characteristics as antecedents of organizational psychological capital: the family involvement in the top management team and the generation of the family firm. We further propose that these relationships are moderated by a board of directors’ tenure. Based on an empirical analysis of listed U.S. family firms, our results confirm a negative relationship between family membership in the top management team and organizational psychological capital. In addition, we find that descendant family firms exhibit higher levels of organizational psychological capital than founder family firms. The results also confirm the moderating role of board tenure. This study works toward a more holistic view of family firm heterogeneity and specifically how different types of family involvement shape a firm’s positive strategic resources.

## Introduction

Organizational psychological capital—that is captured in the dimensions of organizational hope, confidence, optimism, and resilience—has emerged as an influential theoretical lens in the study of positive organizational behavior (Memili et al. 2020, 2014). Organizational psychological capital is the “shared level of positivity and agency among employees” and helps organizations to flourish (McKenny et al. 2013, p. 159). Memili et al. (2013) emphasize that organizational psychological capital is a particular strength of family firms. In addition to its popularity in academia, organizational psychological capital empowers firms in practice to seize opportunities even in the face of challenges (McKenny et al., 2013). Especially in turbulent times, such as during the COVID-19 crisis, it is ever more vital for family firms to ensure their organizational psychological capital. For instance, displaying organizational hope and optimism might re-energize employees in times of pandemic fatigue and help them navigate difficult situations (De Smet et al. 2021). Organizations that focus on workforce resilience and confident behavior can adapt to and thrive during turbulent times (McNulty 2020). Identifying the drivers of organizational psychological capital has become critical to practitioners and scholars alike (Memili et al. 2013).

However, research thus far lacks empirical evidence about why organizational psychological capital is prevalent in family firms. Drawing on Memili et al. (2013) conceptual framework, it is the family firm idiosyncrasies that may foster higher levels of organizational psychological capital. For instance, the family’s involvement in and emotional attachment to the business and their reciprocal altruism can create mutual trust, strong commitment, and high-quality relationships among employees, which “may foster development of unique [organizational psychological capital] of family firms.” (Memili et al. 2013, p. 1291). Yet, family firms are far from being a homogenous group and vary with respect to their characteristics, such as family involvement, power, and experience which might help explain diverging levels of organizational psychological capital (Klein et al. 2005; Villalonga and Amit 2006). Especially the participation of family members in C‑level management can be pivotal to how family firms think and act (Minichilli et al. 2010). In comparison to passive family ownership, active family participation in top management strengthens the family’s identification with the firm, thereby influencing organizational values, attitudes, and decisions (Finkelstein and Hambrick 1990; Hambrick 2007), and most likely also psychological attributes of the family firm. Further, the generation of the family firm shapes its behavior and attitudes. While earlier literature has mostly taken a static view on family firms, more recent works show that family firms evolve over time (Chrisman et al. 2014a; Chrisman et al. 2021). The founder’s influence seems particularly remarkable, and it clearly distinguishes first-generation from later-generation family firms along various dimensions (Gómez-Mejía et al. 2011; Villalonga and Amit 2006).

The purpose of this research is to examine the family-related drivers of organizational psychological capital. Drawing on the concept of socio-emotional wealth (Gómez-Mejía et al. 2007) and following extant research on family firm heterogeneity (Neubaum et al. 2019), this study explores how different forms of family involvement shape organizational psychological capital and focuses on two essential family firm idiosyncrasies: (1) the degree of family involvement in the top management team (TMT), and (2) the generation of the family firm (i.e., descendant-led or founder-led family firms). We further argue that these relationships do not hold universally but expect board tenure to shape how family firm idiosyncrasies relate to organizational psychological capital. Specifically, we synthesize insights of studies on family firms and board characteristics and investigate the moderating role board tenure plays for the link between family involvement and organizational psychological capital. Extant research indicates that particularly experienced boards (i.e., with long board tenure) largely influence family firms’ strategic agenda and managerial decisions (Corbetta and Salvato 2004) and can also affect shared positivity aspects such as organizational psychological capital (Caspersz and Thomas 2013). For instance, board members with longer tenure strengthen social ties among employees, foster employees’ emotional attachment to the family firm, and increase levels of familiarity (Bendig et al. 2020; Le Breton-Miller and Miller 2013; Sun and Bhuiyan 2020; Wilson et al. 2013).

To test our research model on organizational psychological capital in family firms, we use manually collected data on U.S. family firms in the S&P 500 index; the period of observation spans eight years from 2006 to 2013. We contribute to research in at least two ways. First, our notion of family firm idiosyncrasies as antecedents of organizational psychological capital advances the discussion on explaining heterogeneity in family firms as it reveals how shared positivity varies with the extent of family involvement and across generations. Thereby, we offer one of the few empirical studies on how different types of family involvement shape a firm’s positive strategic resources (Chrisman et al. 2014a) and emphasize a novel facet of socio-emotional wealth by discussing how family firm characteristics can also have a detrimental effect on an intangible organizational resource. Second, we enhance the literature by adding board tenure as a relevant contingency influencing the relation between family firm idiosyncrasies and organization psychological capital. Specifically, this puts the role of boards of directors as an important but empirically often overlooked governance body in family firms in perspective as we confirm their importance as a facilitator to fostering levels of organizational psychological capital. Our findings are also important for practice, in particular for family firm leaders and their board of directors. We indicate that high family involvement can restrain the shared level of positivity in firms which should be considered while hiring additional family versus nonfamily top managers.

## Conceptual Background and Hypotheses

Drawing on the resource-based view, Luthans et al. (2004) emphasize that the psychological capital of individual employees, along with human and social capital, constitutes an important source of competitive advantage. Scholars recognize that psychological capital plays not only a pivotal role at the individual but also at the team and organizational level (F Luthans and Youssef-Morgan 2017). Building on social comparison theory, earlier on applied to other psychological phenomena such as moods (Sullins 1991; Totterdell 2000), McKenny et al. (2013) posited that psychological capital spreads through organizations from one person to the next. Specifically, they underlined that regular interactions among employees lead positive psychological resources to converge through social contagion, resulting in a shared level of psychological capital (Clapp-Smith et al. 2009; Dawkins and Sanderson 2015; McKenny et al. 2013). McKenny et al. (2013) proposed psychological capital as a relevant construct in positive organizational behavior research and elevated it from an individual to an organizational level while maintaining its essential building blocks: organizational confidence, hope, optimism, and resilience. Firms with high levels of organizational psychological capital display the following traits: (1) faith in overcoming difficult tasks (*organizational confidence*); (2) belief in achieving corporate goals (*organizational hope*); (3) ability to recover from obstacles (*organizational resilience*); and (4) association of positive events with internal, persistent causes versus the association of negative events with external, context-specific causes (*organizational optimism)*. These four dimensions reflect the “organizational level of positive psychological resources” (McKenny et al. 2013, p. 175).

Translating individual psychological capital to the organizational level, McKenny et al. (2013) also showed the firm-wide shared level of positivity (i.e., organizational psychological capital)to be a significant predictor of financial performance. Their results are the first empirical indication that organizational psychological capital plays an important role as a “strategic organizational resource,” the outcomes and antecedents of which should be further investigated (McKenny et al. 2013). In the context of family firms, Memili et al. (2013) emphasize that family involvement should foster a shared level of positivity in organizations, arguing that families’ socio-emotional embeddedness in firms encourages reciprocal altruism, high-quality relationships, and long-lasting organizational commitment that extends to non-family employees. Family firms are expected to develop a unique organizational flair that promotes organizational psychological capital mainly through family bonding, collectivity, shared history, open organizational culture, and decentralization (Memili et al. 2013).

However, building on the theoretical paradigm of socio-emotional wealth, family firms differ greatly in terms of family-related characteristics, which in turn will likely shape organizational psychological capital differently. Key family-related characteristics in this context are: the extent of ownership and control in the hand of the family (Chua et al. 2012; Klein et al. 2005; Villalonga and Amit 2006); the representation of the family in management positions (Klein et al. 2005; Villalonga and Amit 2006); the family experience measured as the generation in charge of owning, controlling, and managing the business; and the family values as reflected in firm culture (Klein et al. 2005). In this vein, we suggest that it is increasingly important to focus on the heterogeneity of family firms and how it shapes their strategic resources. That way, we can develop a more accurate understanding of how family involvement and the associated socio-emotional wealth contribute to family firms’ uniqueness—and to their organizational psychological capital (e.g., Chrisman et al. 2018; Duran et al. 2016).

### Family Membership in Tmts and Organizational Psychological Capital

The socio-emotional wealth paradigm proposes that the presence of socio-emotional wealth strongly depends on family members’ participation in the TMT. Active family involvement through TMT positions promotes a stronger association of the family name with the firm (Dyer and Whetten 2006) and strengthens the family’s emotional attachment to the business. Increasing family member participation in the TMT translates into rising interest in, and the necessary power to pursue, strategies that help preserve the family’s socio-emotional wealth (Berrone et al. 2010). Empirical evidence shows (Miller et al. 2011): Higher family influence attained through leadership positions promotes utility-maximizing behaviors such as the organizational entrenchment of family members and the pursuit of strategic decisions that serve the family’s affective needs beyond financial considerations. The utility-maximizing behavior of family members may cause severe conflicts of interest with non-family employees—especially if it interferes with attractive expansion or growth strategies, or if it entails increased business risks (Miller and Le Breton-Miller 2014).

External market reactions also reflect the effects of family executives’ utility-maximizing behavior (Granata and Chirico 2010; Villalonga and Amit 2006). Studies have, for instance, found a link between declining stock prices and the appointment of a family member to the CEO position (Smith and Amoako-Adu 1999). Other research reveals that firm values are significantly lower in large family firms in which family members hold leadership positions (Miller et al. 2013). Similarly, family firms with family officers and high family equity stakes appear less valuable to potential acquirers who perceive them as less professionally managed and less efficient (Granata and Chirico 2010). The entrenchment of family members—regardless of their capabilities—and a tendency to pursue strategic decisions out of the desire to preserve socio-emotional wealth rather than economic rationality often characterize family firms (Gómez-Mejía et al. 2007; Miller and Le Breton-Miller 2014). For external markets, high levels of family influence through TMT positions contradict established “logics of objectivity,” and are ultimately perceived as impairing economic robustness (Naldi and Gomez-Mejia 2013, p. 1351).

We expect this negative reaction to family top managers to be reflected not only externally but also internally in form of lower shared positivity (i.e., organizational psychological capital). Extant research on family businesses indicates that a higher share of family members in the TMT might result from nepotistic staffing policies aimed at preserving socio-emotional wealth (Naldi and Gomez-Mejia 2013). However, it is not necessarily in the best interest of the firm if family members hold managerial positions that do not match their capabilities (Naldi and Gomez-Mejia 2013). Family members derive strong emotional satisfaction from promoting their successors to leading positions in the business, but nepotistic staffing policies might substantially reduce shared levels of organizational confidence, optimism, and resilience among employees (Miller and Le Breton-Miller 2014). Lower shared organizational confidence, optimism, and resilience might increase employees’ uncertainty about the ability to achieve organizational objectives by effectively deploying human resources (i.e., organizational confidence); the same applies to the ability to recover from setbacks or failures (i.e., organizational resilience). Family members in the TMT may also be less inclined to externalize negative experiences (i.e., organizational optimism). Moreover, strong bonding ties among family members may impede the emergence of weaker bridging ties to non-family members. Such bridging ties are needed to exchange information effectively and to engage in fruitful strategic discussions aimed at achieving organizational goals (Miller et al. 2011). These unique dynamics may result in lower organizational confidence and optimism as well as in lower levels of overall organizational psychological capital.

A high share of family members in the TMT also entails the risk of incongruent goals pursued by family and non-family stakeholders (Patel and Cooper 2014); this may ultimately harm the shared dedication to organizational objectives (i.e., organizational hope). Research suggests that public family firms with strong family involvement in leadership prefer strategic conservatism and tend to avoid risk to preserve socio-emotional wealth (Chrisman et al. 2014b; Miller et al. 2011; Naldi and Gomez-Mejia 2013). These preferences often counter the interests of non-family stakeholders, who tend to be more risk-inclined and willing to invest even in uncertain growth opportunities. Incongruent goals of family and non-family stakeholders may weaken firm members’ belief that organizational goals are achievable (i.e., organizational hope). Collectively, the implications of a high share of family members in the TMT likely contribute to a decline in organizational psychological capital. We thus hypothesize:

#### Hypothesis 1:

Family TMT membership negatively relates to organizational psychological capital in family firms.

### Family Firm Generation and Organizational Psychological Capital

Gómez-Mejía et al. (2007) point out that the relative importance of socio-emotional wealth changes with the company transitioning from one generation to the next. Family firms develop over time and across generations, and the differences between founder- and later-generation family firms are likely decisive (Gómez-Mejía et al. 2011). The founding family’s socio-emotional endowments are highest in first-generation family businesses. As founders, family members tend to identify closely with their firms, and they derive strong social status from their firms (Gómez-Mejía et al. 2011; Miller and Le Breton-Miller 2014). They often maintain high levels of organizational control and preserve their socio-emotional endowment priorities (Chua et al. 1999; Gómez-Mejía et al. 2007). As family descendants take over, socio-emotional endowments in the business may gradually decline (Gómez-Mejía et al. 2007): emotional ties and personal identification diminish, equity stakes become more and more dispersed—all of which shifts family firms’ focus away from preserving socio-emotional endowments towards accomplishing optimum economic performance (Chua et al. 1999).

Empirical studies highlight the differences between founder and descendant family firms (Brun De Pontet et al. 2007; Gómez-Mejía et al. 2007; Villalonga and Amit 2006). Scholars show descendant generations to be less reluctant than founder generations to decentralize control if it enhances economic performance (Brun De Pontet et al. 2007; Gómez-Mejía et al. 2007), and they more readily apply formalized human resource management practices or control systems (Gómez-Mejía et al. 2001; Reid and Adams 2001). Descendants rely less on nepotistic personnel policies, evaluate family members along strict criteria of competence, and are more likely to let go family executives not meeting performance standards (Gómez-Mejía et al. 2001). Accordingly, alignment and affiliation with non-family organizational members should increase as family firms transition from one generation to the next.

We posit that the generation-to-generation evolution of family firms fosters organizational psychological capital. Descendant family firms that adopt structured personnel policies and strategic decision-making processes prioritizing economic growth should develop higher levels of shared positivity. Strict, objective evaluation of family members’ performance is expected to enhance both shared levels of confidence that the firm has the right human resources to achieve its organizational objectives (i.e., organizational confidence) and the ability to manage challenging situations and recover from setbacks (i.e., organizational resilience). Moreover, growing confidence in family firms’ own resources often translates into a stronger tendency to externalize negative situations or failures (i.e., organizational optimism). We also expect that organizational hope increases as descendant family firms focus less on socio-emotional objectives and more on economic rationality. Patel and Cooper (2014) find a decline in conflicts of interest when family firms transition from one generation to the next. We argue that family and non-family organizational members who increasingly act in concert are likely to experience enhanced commitment and a strong belief in achieving organizational goals and objectives (i.e., organizational hope). We suggest that higher levels of organizational psychological capital emerge in family firms in which family members of the second, third, or later generation participate in ownership, top management, and/or the board of directors.

#### Hypothesis 2:

Family firm generation positively relates to organizational psychological capital, higher levels being observed in descendant than in founder family firms.

### The Moderating Role of Board Tenure

The socio-emotional wealth perspective assumes that the board of directors’ role in protecting and maintaining a family firm’s socio-emotional wealth and “in deciding upon strategy” is a central one (Wilson et al. 2013, p. 1369). The board intensively interacts with the family members in the family firm and plays a crucial part in fostering a sense of familiarity (Huang and Hilary 2018) and collectivity among employees (Barroso-Castro et al. 2016), both of which might help shape organizational psychological capital. Besides the TMT, the board constitutes a family firm’s most important information-processing group (Schepker et al. 2018), transforms newly acquired information disseminated throughout the organization into performance outcomes (Barroso-Castro et al. 2016), and determines as well as monitors the implementation of the strategic agenda (Corbetta and Salvato 2004).

Studies on board characteristics in family firms find that board tenure is particularly important in preserving social ties and emotional attachments, which might influence the relationships between family firm idiosyncrasies and organizational psychological capital (H1 and H2) (Le Breton-Miller and Miller 2013; Wilson et al. 2013). Longer board tenure fosters board stability, which, in turn, increases familiarity and decreases conflicts in family firms (Sun and Bhuiyan 2020). Moreover, board tenure promotes smooth information flows and knowledge diffusion and transformation across the organization (Nahapiet and Ghoshal 1998). We suggest that experienced board members (i.e., with longer tenure) might help lead and manage organizational positivity (Caspersz and Thomas 2013). Put more precisely, we expect longer board tenure to weaken the negative effects of a high family share in the TMT and to strengthen the positive effects that emerge in descendant-led firms.

A board consisting of long-tenured members has the potential to counteract the negative effects of higher degrees of family involvement in the TMT for the following reasons. First, extant research reveals that board members’ time invested in interacting with colleagues frequently forms the foundation of board-internal social capital (Barroso-Castro et al. 2016). The board’s internal social relationships encourage family firm employees to develop “trust, norms, and identity, as well as to believe in a common vision and purpose, […] expressed in unique language and ideas” (Pearson et al. 2008, p. 958). As a result, the shared vision and language created by increased board tenure serve as bonding mechanisms that foster strong relationships. Hence, we suggest that a long-tenured board is helpful in creating a working environment that counteracts the negative effects of high family involvement in the TMT, which is, in turn, fruitful for organizational hope and resilience. Second, strong internal network ties, resulting from tenure, strengthen collective goals and cohesiveness (Adler and Kwon 2002), “reduce transactions costs, [and] facilitate information flows, [and] knowledge creation and accumulation” (Arregle et al. 2007, p. 73). Shared goals are fruitful in boosting organizational hope and optimism. In fact, long-tenured boards often hold high-quality, trustful, respectful relationships with employees (Cuevas-Rodríguez, Cabello-Medina, and Carmona-Lavado 2014), which has the chance to weaken the negative effects of family involvement on family firms’ level of organizational psychological capital. Third, the board of directors, as a crucial governance body, has the legal duty of “planning leadership succession” (Umans et al. 2020, p. 190). As prior research indicates, some family firms might be subject to nepotistic staffing policies (Naldi and Gomez-Mejia 2013) or to incongruent staffing goals of family and non-family stakeholders (Patel and Cooper 2014). Especially in the context of family firms with higher family involvement in the C‑suite, against which there are prejudices concerning nepotistic staffing policies, a long-tenured board might provide benefits to organizational confidence and optimism. We consequently hypothesize that increased board tenure attenuates the negative relationship between family TMT involvement and organizational psychological capital.

#### Hypothesis 3a:

The negative relationship between family TMT membership and organizational psychological capital is attenuated when board tenure is high and more pronounced when board tenure is low.

In Hypothesis 2, we propose that the levels of organizational psychological capital are higher in descendant than in founder family firms. We now argue that board tenure positively moderates this relationship. Boards with long tenure allow to retain cognitive, relational, and structural social capital “through tacit knowledge routines” (Steier 2001, p. 274) characterized by stability based on lasting and durable relationships, in some cases even over family firm generations (Pearson et al. 2008). Experienced and tenured boards should be well equipped to support family firms in transferring internal social networks from one generation to the next (Steier 2001). Family firms successfully fostering internal social networks over time and across generations can benefit from long-standing relationships deeply rooted in the family tradition as well as from new relational ties (Steier 2001). We expect an experienced board, expressed in increased tenure, to facilitate family firms’ ability to address challenging situations and recover from setbacks (i.e., organizational resilience). We also presume that tenured boards promote shared commitment towards firm goals and objectives (i.e., organizational confidence and optimism) across generations.

The process of generational succession comprises social exchange. For instance, boards help nurture and develop successors through incumbent/successor, across-family-boundary, and within-family-boundary exchanges, among other forms of social exchange (Daspit et al. 2016). Experienced and tenured boards forward the success of succession plans given the profound and trustful relationships among family members, non-family members, and even key customers or suppliers (De Massis et al. 2008). We expect that such relationships also enhance a family firm’s level of organizational psychological capital because the inherent trust bolsters a strong belief in achieving organizational goals and objectives (i.e., organizational hope). While planning a succession, family firms usually consider many different stakeholder perspectives—this underscores that the firm can draw on suitable human resources to achieve its organizational objectives (i.e., organizational confidence). Cabrera-Suárez (2005) finds that supportive, loyal, collaborative non-family managers are a common feature of successful succession processes. Assuming that such positive managers within the board strengthen the overall level of positivity in family firms, we hypothesize that board tenure enhances the positive relationship between descendant family firm generation and organizational psychological capital.

#### Hypothesis 3b:

The positive relationship between descendant family firms and organizational psychological capital is strengthened when board tenure is high and weakened when board tenure is low.

## Methods

### Sample and Data Collection

Our empirical analyses rely on a panel dataset comprising U.S. family firms across industries listed in the S&P 500 index. We followed previous research in defining family firms as firms in which family members have equity ownership and/or occupy a seat in the director’s board (Anderson and Reeb 2003; Barth et al. 2005; Cronqvist and Nilsson 2003). Banks and public utility firms were excluded due to regulatory peculiarities (Anderson et al. 2012; Lee 2006). Based on this definition, we initially identified 258 family firms. To ensure high comparability of the family firms across time, we included only firms that were continually listed in the S&P 500 Index across all panel years (Short et al. 2009).

We manually compiled information on the family firms from annual reports and merged it with data gleaned from letters to shareholders, proxy statement information, and the Compustat North America database. In line with McKenny et al. (2013), we analyzed firms’ letters to shareholders with computer-aided text analysis to measure organizational psychological capital. Such letters in annual reports communicate to shareholders a firm’s most salient organizational themes at the time of publication (McKenny et al. 2013; Segars and Kohut 2001). Given that the Sarbanes-Oxley law requires managers to certify the accuracy and completeness of annual reports, letters to shareholders represent verified and endorsed information (McKenny et al. 2018). Such texts provide top managers with a vehicle for conveying their firms’ degree of shared positivity to investors and are particularly valuable for evaluating organizational psychological capital.

To investigate the heterogeneity of family firms that results from variances in family influence, we expanded our dataset with detailed information on the degree of family involvement in the sample firms. We searched proxy statements, annual reports, and company websites for data on family ownership shares, family involvement in TMTs and boards of directors, and owning, controlling, and managing generation. We completed our dataset with financial statement information from the Compustat North America database. The derived sample includes only firm-years with complete data for all variables. For our estimation, we included only observations for which we had data for two consecutive years, resulting in 340 firm-year observations (57 family firms).

### Measures

#### Organizational Psychological Capital

To measure organizational psychological capital, we employed computer-aided text analysis to determine the prevalence of constructs based on the frequency of words in a text (McKenny et al. 2018)—letters to shareholders, in our case. This method essentially builds on the Whorf-Sapir Hypothesis, which proposes that the frequency of a word’s usage indicates its level of attention (Whorf 1956). Computer-aided text analysis, because it does not rely on subjective (human) assessment based on a few respondents, affords a more objective approach to measuring organizational psychological capital.

McKenny et al. (2013) developed and validated a list of words for each dimension of organizational psychological capital. For example, words associated with confidence include ability, accomplish, and conviction. To count the number of words associated with the four dimensions in each of the letters to shareholders, we followed McKenny et al. (2013) in using the CAT Scanner software, which is particularly advantageous when working with large volumes of texts. For each letter, we calculated the number of words related to organizational confidence, hope, optimism, and resilience with the CAT Scanner software. Subsequently, we added the number of words associated with the respective dimension and divided this sum by the total words to account for different text lengths. Based on this calculation, we derived a relative share of words associated with organizational psychological capital in each of the letters to shareholders. For example, an organizational psychological capital score of 0.022 indicates that 2.2% of all words in a letter relate to organizational psychological capital. This approach follows McKenny et al. (2013), who introduced organizational psychological capital as a superordinate construct based on its four lower-order dimensions: organizational confidence; organizational hope; organizational optimism; and organizational resilience. Before summing the scores of each organizational psychological capital dimension, we closely examined the dimensionality of the construct. In line with McKenny et al. (2013), we looked at the correlations among organizational confidence, hope, optimism, and resilience to assess whether any of the dimensions needed to be combined. None of the correlations exceeded 0.4, which confirms using the four dimensions for measuring organizational psychological capital (McKenny et al. 2013; Short et al. 2010).

We assessed the degree of potential measurement error variance in our computer-aided text analysis. Again following McKenny et al. (2018), we focused on the assessment of algorithm error, transient error, and specific factor error as the three most common sources of measurement error in computer-aided text analysis constructs. We found none of these errors to threaten our organizational psychological capital variable (Table 1). We first tested the degree of algorithm error to assess the variance that might arise from using two different software packages (i.e., CAT Scan and LIWC). We remeasured organizational confidence, hope, optimism, and resilience using LIWC 2015 as an alternative word-count analysis program and measured its variance with our original score based on the CAT Scanner software. The results showed the percentage of the variance between the two software packages to be only 1% (mean Krippendorff’s alpha = 0.99). The CAT Scanner thus assures strong measurement accuracy, accounting, for example, for special characters in the analyses that could bias the results. We next investigated fluctuations over time in our organizational psychological capital score by averaging the correlation for each pair of consecutive years (McKenny et al. 2018). Results showed the percent of variance due to transient error to be relatively high, with an average correlation of 0.46. Based on the underlying theory, this is not surprising given that organizational psychological capital is a state-like construct that develops over time (Fred Luthans 2002). Indeed, McKenny et al. (2018) pointed out that temporal variance is particularly likely to occur with state-like constructs such as organizational psychological capital. As our sample includes the years of the financial crises, we acknowledge that exogenous shocks might account for changes in organizational psychological capital (McKenny et al. 2018).

**Table 1:** Results of Measurement Error Assessment for Computer-Aided Text Analysis Measures of Organizational Psychological Capital

Error Source	Test of Reliability	Mean Reliability Estimate
Algorithm error	Krippendorff’s alpha	0.99
Transient error	Test-retest	0.46
Specific factor error	Parallel forms	0.83

Lastly, we assessed variance due to specific factor error by manually coding a subsample of our letters to shareholders. Two research experts manually coded 104 randomly selected letters to shareholders. Correlating the organizational psychological capital scores derived from the manual coding process with the scores derived from the CAT Scanner software, we find average parallel forms reliability to be 83% (r = 0.83), which exceeds the 0.8 benchmark set by Nunnally and Bernstein (1994). We thus conclude that the organizational psychological capital measurement is suitable for our research.

#### Family TMT Membership

To assess the share of family members in the TMT, we first identified the family firms among all listings continually present in the S&P 500 Index from 2005 to 2013. Following previous research, we defined family firms as those in which family members hold an equity stake in the firm and/or occupy seats on the board of directors (Anderson and Reeb 2003; Barth et al. 2005; Cronqvist and Nilsson 2003). Similar to previous S&P 500 family firm research (Anderson and Reeb 2003), we identified 28% of all firms as family firms, 85% of which had family members on the board of directors. Next, we collected information on the relative share of family members in the TMT. In line with previous research, we measured family representation in the TMT as the share of family versus non-family member seats in the TMT (González-Cruz and Cruz-Ros 2016). Measured on a continuous scale, the variable takes a value between 0 (i.e., no family members in the TMT) and 1 (i.e., only family members in the TMT).

#### Family Generation

To differentiate between first-generation (i.e., founder) and descendant family firms, we defined in our model a binary variable to which a value of 0 was assigned if the founder generation was active in the business. In contrast to Miller et al. (2007), who classify as first-generation family firms only firms with multiple first-generation family members, we followed Villalonga and Amit (2006) in requiring at least one founding family member to be involved in the business through an equity stake and/or position on the board of directors and/or TMT. For all other family firms with second-, third-, or later-generation family members involved in the business, our binary variable took the value of 1. Firms including both founder and descendant generation family members were classified as descendant family firms. We expect the descendant generation to have a discernible effect on the business that differentiates them from family firms in which only the founder generation is involved.

#### Board Tenure

We assessed board tenure by calculating the average tenure of all board members per firm at the time. Higher average tenures of board members reflect long-term experiences that foster a sense of familiarity and collectivity (Barroso-Castro et al. 2016; Huang and Hilary 2018). Information on the tenure of each director was gleaned from yearly data on board composition obtained from proxy statements, annual reports, and company websites.

#### Control Variables

We included relevant firm and environmental control variables in our study. At the firm level, we included *firm size*, which might also be an important predictor of organizational psychological capital because it can profoundly influence family firms’ governance structure (Bammens et al. 2008) and behavior (Gómez-Mejía et al. 2011). We measured firm size as the logarithm of total sales (Berrone et al. 2010). We next accounted for the previous year’s *market capitalization, *measured as the share price multiplied by outstanding shares, and last year’s *book-to-market value, *calculated as the difference between current assets and liabilities divided by market capitalization. Both variables, being valuable indicators of firm performance (Hawn and Ioannou 2016; Miller et al. 2007), might affect the shared level of positivity in family firms. To account for outliers, we winsorized firm size at the 5% level and market capitalization and book-to-market value at the 1% level. We further included *profit volatility* in the past two years, widely used by previous scholars as a predictor of financial health (Holt et al. 2017; Zellweger et al. 2012). Because high variations in firm profit might intensify feelings of uncertainty and reduce organizational psychological capital, we controlled for profit volatility effects in our model. Specifically, we calculated each firm’s profit volatility based on profit margin deviations between t‑2 and t. We also added *labor intensity* and* effort intensity, *which indicate the extent of variable resources in a firm (Bantel and Jackson 1989; Dotzel et al. 2013) and influence firm structures and processes. Following previous research, we operationalized labor intensity as employees divided by total sales in USD millions (Swink and Jacobs 2012) and effort intensity as cost of goods sold divided by total sales, both measured in USD millions (Dotzel et al. 2013). We also accounted for important dynamics in firms’ competitive environments and market positions. Scholars have pointed out the influential role of *competitive intensity*: firms in dynamic, competitive environments are exposed to higher uncertainty (Boyne and Meier 2009; Fred Luthans 2002). To control for this effect, we used the Herfindahl-Hirschman index, calculated as the sum of the squared market share (based on sales) of all firms in one industry (Saboo et al. 2016). Research establishes firm market share as a positive predictor of firm outcomes (Luthans et al. 2007); we also included the control variable firm *market share*, calculated as sales divided by industry sales. To account for variance in firms’ shared level of positivity due to market growth, we assessed *market demand growth*, measured as the annualized average industry sales growth in the past three years (Morgan and Rego 2006). We calculated environmental control variables based on firms’ 3‑digit standard industrial classification (SIC) code. Lastly, we controlled for year-specific effects by including *year dummies* in our model.

### Modeling

We apply generalized estimation equation modeling (GEE) to test our hypotheses empirically. GEE models are maximum likelihood models that are particularly valuable in time-series studies because they do not assume independence of observations over time (Ballinger 2004). They allow us to specify an autoregressive within-group correlation structure with robust standard errors to account for the non-independence of observations of family firms across the sampling frame. With the Pan’s quasi-likelihood information criterion (QIC) indicating that it was the best fit for our model, we set an autoregressive one-year within-subject correlation structure.

To achieve normality, we transformed our organizational psychological capital score by taking its square root. As a post-transformation Shapiro-Francia test revealed our response variable to be normally distributed (V’ = 0.50; *p* = 0.94), we specified in all our models a Gaussian distribution with an identity link function. Prior to the regression analyses, we standardized all variables.

## Results

Table 2 reports descriptive statistics and correlations. In line with our hypotheses, the results indicate that family TMT membership is negatively correlated with organizational psychological capital (r = −0.20, *p* < 0.001), whereas generation of family firm is positively correlated with organizational psychological capital, with the latter correlation being statistically insignificant (r = 0.02, *p* > 0.10). Correlations between all remaining variables are low to moderate. The individual variance inflation factors in our model range from 1.12 to 2.21 and do not indicate multicollinearity, although variance inflation factors should be interpreted with caution and cannot rule out multicollinearity (Kalnins 2018).

**Table 2:** Means, Standard Deviations, and Correlations

Variables		μ	SD	1	2	3	4	5	6	7	8	9	10	11	12	13
1	Organizational psychological capital ^a^	2.44	0.78	1	–	–	–	–	–	–	–	–	–	–	–	–
2	Family TMT membership	0.11	0.14	−0.20***	1	–	–	–	–	–	–	–	–	–	–	–
3	Family generation	0.53	0.50	0.02	−0.10	1	–	–	–	–	–	–	–	–	–	–
4	Board tenure	0.07	1.00	0.03	0.06	−0.19***	1	–	–	–	–	–	–	–	–	–
5	Firm size (ln)	8.98	0.60	−0.12*	0.03	0.17 **	−0.23***	1	–	–	–	–	–	–	–	–
6	Market capitalization	23.21	18.78	−0.14**	−0.14**	−0.02	−0.15**	0.52***	1	–	–	–	–	–	–	–
7	Book-to-market value	0.36	0.33	0.00	0.09	−0.04	0.05	0.07	−0.15**	1	–	–	–	–	–	–
8	Profit volatility	0.04	0.07	0.13*	−0.04	−0.11*	0.02	−0.15**	−0.04	0.28***	1	–	–	–	–	–
9	Labor intensity	0.00	0.00	0.25***	0.04	−0.18**	0.03	0.00	−0.17**	−0.13*	−0.18***	1	–	–	–	–
10	Effort intensity	0.53	0.23	−0.24***	0.24***	0.18***	−0.02	0.44***	−0.05	0.24***	−0.11*	−0.03	1	–	–	–
11	Competitive intensity	1.28	1.04	0.12*	−0.12*	0.08	0.10	−0.29***	0.02	−0.11*	0.16**	−0.26***	−0.33***	1	–	–
12	Market share	0.21	0.26	0.05	0.01	−0.10	0.14**	0.31***	0.18***	−0.07	−0.25***	0.18**	0.37***	−0.36***	1	–
13	Market demand growth	0.05	0.08	0.00	−0.04	0.01	0.01	0.15**	0.20***	−0.16**	−0.06	−0.07	−0.15**	0.05	−0.10	1

^a^ Words associated with organizational psychological capital indicated in % (μ; standard deviation)

*Notes.* N = 340 (57 firms); SD = standard deviation; *** *p* < 0.001; ** *p* < 0.01; * *p* < 0.05

Table 3 contains the results of our main GEE models (Models I–VI). Hypothesis 1 predicts family membership in the TMT to relate negatively to organizational psychological capital in family firms. The results in Model II show the coefficient of family TMT membership to be negative and significant (β = −0.15, *p* < 0.01), supporting Hypothesis 1. Model III investigates the association between generation of family firm and organizational psychological capital. As expected, we find descendant (i.e., second-, third-, or later-generation) family firms to have higher levels of organizational psychological capital than founder family firms (β = 0.28, *p* < 0.10). Hypothesis 2 is supported at the 10%-level.

**Table 3:** GEE Regression Results Assuming Autoregressive One-Year Correlation (Dependent variable: Organizational psychological capital)

Independent variables	Model I (Base)	Model II (H1)	Model III (H2)	Model IV (H3a)	Model V (H3b)	Model VI (H1–H3b)
Firm size	0.14	0.15	0.11	0.16†	0.10	0.12
Market capitalization	−0.27**	−0.28***	−0.25**	−0.28***	−0.25**	−0.26**
Book-to-market value	0.08	0.08	0.09†	0.08†	0.08	0.08†
Profit volatility	0.19*	0.18*	0.21**	0.18*	0.20**	0.19*
Labor intensity	0.30**	0.31***	0.33**	0.31***	0.34***	0.35***
Effort intensity	−0.35***	−0.32***	−0.37***	−0.34***	−0.38***	−0.37***
Competitive intensity	0.19*	0.18*	0.17*	0.18*	0.16*	0.15†
Market share	0.24**	0.22**	0.26**	0.21*	0.28***	0.26***
Market demand growth	0.08	0.07	0.08	0.07†	0.10†	0.09†
Board tenure	−0.06	−0.04	−0.04	−0.05	−0.18*	−0.17**
Family TMT membership	–	−0.15**	–	−0.14**	–	−0.12*
Family generation	–	–	0.28†	–	0.27*	0.24†
Family TMT membership × Board tenure	–	–	–	0.08†	–	0.08†
Family generation × Board tenure	–	–	–	–	0.32*	0.30*
*N*	340	340	340	340	340	340
Wald-Chi^2^	80.37	88.73	76.37	99.18	80.01	110.11

*Notes.* N = 340 (57 firms); Standardized regression coefficients are reported for non-dummy variables; year controls are included but not reported

*** *p* < 0.001; ** *p* < 0.01; * *p* < 0.05; † *p* < 0.10

**Fig. 1:**
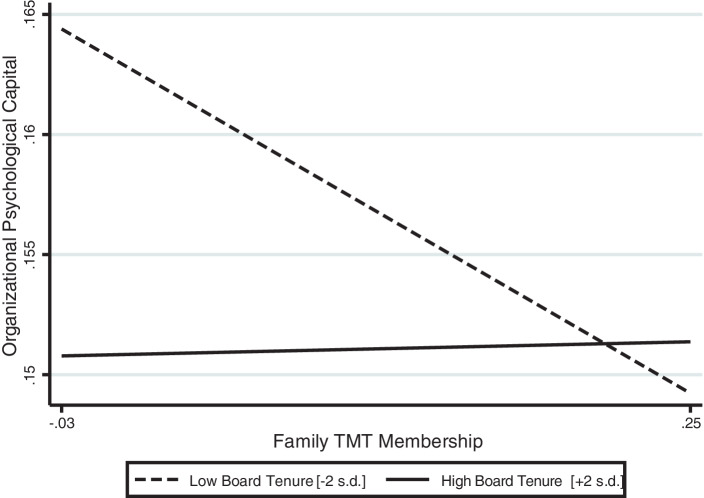
Moderating Effect of Board Tenure on the Relationship between Family TMT Membership and Organizational Psychological Capital

**Fig. 2:**
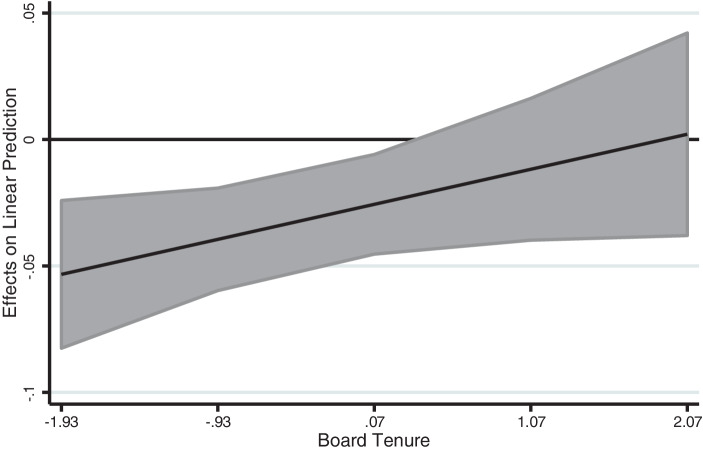
Average Marginal Effects of Family TMT Membership (with 95% Confidence Intervals)

Models IV–V include board tenure and the corresponding interaction terms. Hypothesis 3a argues that high board tenure attenuates the negative relationship of family influence and organizational psychological capital. The results of Model IV (β = 0.08, *p* < 0.10) do, indeed, show a positive relationship between the interaction term and organizational psychological capital, which supports Hypothesis 3a. Estimating a simple slope test shows how the margins differ across ranges (i.e., average marginal effects). Keeping family TMT membership constant, we can derive that an increase in board tenure (+2 standard deviations (s. d.) above the mean) is associated with a decrease of the negative relationship of family membership in TMTs and organizational psychological capital. The probability is then displayed with 0.01%, whereas the probability of a negative relationship with lower levels of board tenure (−2 s. d. below the mean) is 5%. Further, we consider different levels of TMT membership and board tenure. The predicted value of organizational psychological capital is 0.15 in case family TMT Membership is +1 s. d. above the mean (0.25) and board tenure +2 s. d. above the mean (2.07). Figs. 1 and 2 display these effects. Also, the slope is significantly different from zero when board tenure is 1 s. d. below the mean (*p* < 0.01), but insignificantly different from zero when board tenure is 1 s. d. above the mean (*p* > 0.1).

**Fig. 3:**
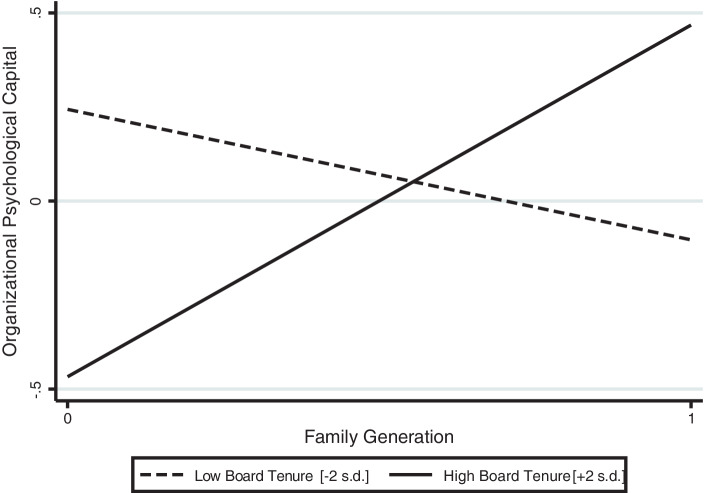
Moderating Effect of Board Tenure on the Relationship between Family Generation and Organizational Psychological Capital

**Fig. 4:**
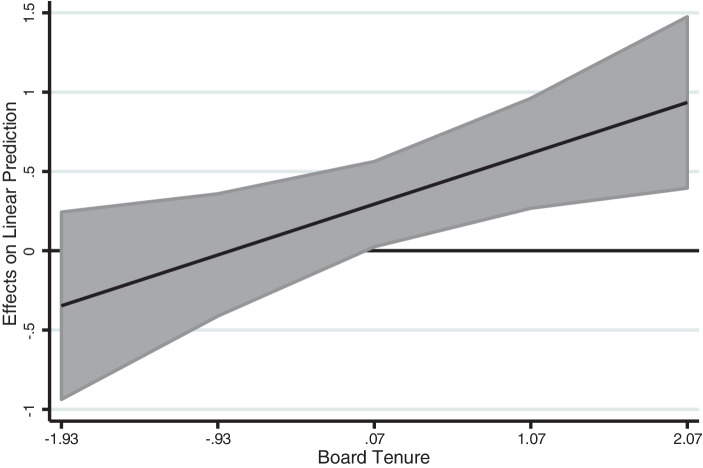
Average Marginal Effects of Family Generation (with 95% Confidence Intervals)

Hypothesis 3b predicts high board tenure to increase the positive effects of descendant family generation on organizational psychological capital. This hypothesis is also supported, as Model V shows (β = 0.32, *p* < 0.05). The full model (Model VI) confirms all results. The estimation of margins prediction by high levels of board tenure (+2 s. d.) and descendant family firm shows a 47% increase in organizational psychological capital. Figs. 3 and 4 show the interaction and its average marginal effects. Since family firm generation is a binary variable, we used a joint test for slope difference which revealed that the slopes for descendant family firm and founder family generation are significantly different (F = 6.36, *p* = 0.01).

### Robustness Tests

We employed different tests to ensure the robustness of our results. First, we estimated alternative specifications. We reran our estimations using as our response variable the organizational psychological capital scores derived with the LIWC 2015 software. The results remain fully stable. Additionally, recognizing that family members might strongly influence their firms through positions on the boards of directors, we added the ratio of family members on the board of directors to the ratio of family members in the TMT to define total family influence. The results of Hypothesis 1 hold (β = −0.15, *p* < 0.01).

Second, to rule out multicollinearity, we re-estimated our regressions without including firm size as a control variable in our model, which showed moderate correlations with both variables market capitalization (r = 0.52 and effort intensity (r = 0.44)). The direction and significance of our results remained fully stable. We also included squared terms for family TMT membership to assess a potential non-linear relationship. The squared term being statistically insignificant, we stick with the linear relationships in our main model.

Third, we used the hybrid model approach to ensure that the within-cluster effects are not statistically different from the between-cluster effects (Schunck and Perales 2017). Within-family firm or between-family firm effects could reveal different results on organizational psychological capital (Allison 2005). Our main analysis followed a between-firm relationship since we assume that family firms differ with respect to our independent variables over time (Certo and Semadeni 2017). Using the hybrid model approach revealed that the within-cluster effects are not statistically different from the between-cluster effects (Schunck and Perales 2017). Employing STATA “xthybrid”-command, the test of the random effects assumptions on family TMT membership, family generation, board tenure, the respective interaction terms, and controls are not significant (*p* > 0.5).

Fourth, we employ the impact threshold of a confounding variable (ITCV) approach to address potential omitted variable bias. Following prior work (Busenbark et al. 2021; Frank 2000; Frank et al. 2013), we estimate the ITCV for our independent variables family TMT membership and family firm generation. The percent bias needed to invalidate the inference for family TMT membership is 37.66% (128 cases). The partial correlation is 0.22 with organizational psychological capital and −0.22 for family TMT membership. The ITCV for family TMT membership is reflected by −0.05 (=0.22 × −0.22). Comparing the impact of the covariates, market capitalization displays the strongest impact of 0.02 (market capitalization; partial correlations), which implies that an omitted confounding variable necessary to invalidate the inference would require a much larger impact. Further, the percent bias needed to invalidate the inference for family firm generation is 13.77% (47 cases). The partial correlation is 0.08 with organizational psychological capital and 0.08 for family firm generation; the ITCV is displayed at 0.01 (=0.08 × 0.08), which is comparable to the impact values of the other covariates. The strongest control variable displays an impact of 0.03 (market capitalization; partial correlations). Based on the results of the ITCV approach, it appears unlikely that a confounding variable biases the estimates.

## Discussion

Do varying degrees of family involvement in family firms have an impact on organizational psychological capital, a psychological resource that describes the level of organizational confidence, hope, optimism, and resilience? Our study sheds light on family firms’ organizational psychological capital. Its findings provide empirical support for Memili et al.’s (2013) hypothesis on the role of organizational psychological capital in family firms. We reveal family membership in the TMT to negatively relate to organizational psychological capital. Our results show levels of positivity to be lower in family firms with increased family dominance than in those with a lower share of family members in the TMT. High board tenure attenuates this negative relationship. Adopting a developmental perspective on the family firm, we find significant differences in organizational psychological capital based on the family generation. Organizational psychological capital is higher after family firms have transitioned from the founder to descendant generations. We observe a positive moderating effect of board tenure on the relationship between family generation and positivity. Our findings provide both academia and practice with insights into family firm heterogeneity.

### Theoretical Implications

Our study yields new empirical insights and advances the family firm literature in four ways. First, our study expands existing theorizations on the concept of socio-emotional wealth, which increasingly forms the backbone of family firm research. The socio-emotional wealth perspective rests on the assumption that family members, as the dominant principals of the firm, largely direct their choices towards preserving their socio-emotional endowments in the firm. A core aspect is the perpetuation of the family’s control and authority over the business and the pursuit of kinship successions to maintain the family’s close identification with and emotional attachment to the business (Gómez-Mejía et al. 2007). While Memili et al. (2013) theorize that these idiosyncrasies foster organizational psychological capital through reciprocal altruism and high-quality relationships extending to non-family employees, our study indicates the opposite. Highlighting the dark side of socio-emotional wealth, we observe that family TMT membership has a detrimental effect on the development of organizational psychological capital. Earlier studies argue that family involvement and socio-emotional wealth decrease both proactive stakeholder engagement (Kellermanns et al. 2012) and organizational commitment of non-family employees (Patel and Cooper 2014). Similarly, our results underline as well that socio-emotional wealth may not be universally beneficial.

Second, our study sheds light on unique family firm characteristics and offers valuable insights for theories on family businesses. Untangling family firm peculiarities is crucial to developing a better grasp of family firm idiosyncrasies. For instance, we highlight how two different characteristics of family firms can lead to various degrees in organizational psychological capital. Our study shows that organizational psychological capital decreases with a rising share of family members in the TMT, but increases as family firms move from founder to descendant generations. The results also advance Memili et al. (2013) proposition that family firms’ idiosyncrasies (e.g., the perpetuation of family authority, kinship succession) should influence organizational psychological capital, indicating that this resource is not universally shared at the same level. As Chua et al. (2012) have already proposed, differences among family firms may be as large or even larger than distinctions between their family and non-family counterparts. Melin and Nordqvist (2007) even warn against being blind to the heterogeneity of family businesses. Our results corroborate the assertion that a one-size-fits-all logic falls short when trying to fully understand resource setups in family firms. Our study advances this large stream of family firm research and highlights the heterogeneity among family firms by exploring how organizational psychological capital varies with the extent of family involvement and across generations.

Third, we contribute to research on positive organizational behavior. A number of researchers show psychological capital to be an important intangible resource with the power to affect behavior and performance positively at the individual, group, and organizational levels (Avey et al. 2009; Fred Luthans et al. 2008; McKenny et al. 2013; West et al. 2009). However, psychology research still largely focuses on avoiding or treating the negative, not on promoting the positive (Caspersz and Thomas 2013). Two firms, which were not in our sample, illustrate the difference between avoiding the negative and promoting the positive in business practice. During the COVID-19 pandemic, the company LinkedIn gave its workforce a paid week off to avoid burnout (Vasel 2021). The company Cisco strategically incorporates the power of positivity. The company, which ranks number one on Fortune’s 2020 list of the world’s best workplaces, established the Cisco Mindset program to help its workforce develop resilience (Hougaard 2021). Cisco Chief Executive Officer Chuck Robbins and Chief People Officer Francine Katsoudas strongly commit to fostering organizational resilience (CNBC 2020). We argue that it is crucial for firms to shift their interest “from repairing what is broken to nurturing what is best” (Meyers et al. 2013, p. 618). Drawing on positive psychology and positive organizational behavior research, our study advances our understanding of family firms’ internal psychological foundations and provides explanatory power with respect to the idiosyncrasies of family firms (De Massis and Foss 2018).

Fourth, we find that board tenure moderates the link between family firm characteristics and positivity. Introducing board tenure as a moderator increases our understanding of how a family firm’s central governing body shapes organizational psychological capital. Our results confirm that the generation of the family firm as well as the family representation at the apex of the firm and the tenure of the boardroom interact, and are hence ideally examined in combination. The increase in organizational psychological capital in family firms with strong board tenure highlights the need to maintain and develop internal social network ties. With this, we also contribute to recent research on board tenure (Sun and Bhuiyan 2020) and board compositions in family firms (Le Breton-Miller and Miller 2013; Wilson et al. 2013).

### Practical Implications

Family firms represent the most common organizational form worldwide and account for 70% of the global gross domestic product (De Massis et al. 2018). Considering their tremendous economic and societal importance, it is important for practitioners to extend their knowledge of how family firm resource setups evolve. Our results may encourage family firms to explore how to increase levels of organizational psychological capital in the long term. Our study also calls attention to the dark side of a high share of family members in the TMT. It is a question not so much of whether family executives are competent, but rather of what involving family executives implies for the organization. Having many seats in the TMT enables families to influence the organization decisively and to preserve their socio-emotional wealth. Our results, however, indicate that high family involvement can also lower the shared level of positivity in organizations, an important intangible resource that enables firms to flourish. Families with multiple executive positions should probably concurrently introduce measures aimed at protecting and fostering organizational positivity. In this regard, our research highlights the importance of relational wealth. We show how tenured boards act as social catalysts that can improve organizational psychological resources. In addition to family members in the C‑suite, family firms may want to cultivate boards that have strong social ties within the firm and hold their board position for years.

Our study also reveals founder and descendant family firms to display significant variance in the prevalence of organizational psychological capital. The results corroborate previous insights that family firms undergo a profound shift between founder and subsequent generations. Successor generations are often criticized for contributing little to the formation of values (Klein, Astrachan, and Smyrnios 2005) and for viewing the family firm as source of personal income (Block 2012). We, in contrast, disclose that descendant generations may add positive value by enhancing organizational psychological capital. This finding is of particular interest to family firms as earlier research offers empirical evidence that organizational psychological capital represents a valuable strategic resource fostering financial performance (McKenny et al. 2013). To strengthen organizational psychological capital, founder firms might engage in positive psychology interventions specifically designed for family firms (Seligman et al. 2005; Sin and Lyubomirsky 2009). Positive psychology interventions include “any intentional activity or method (training, coaching, etc.) based on (a) the cultivation of valued subjective experiences, (b) the building of positive individual traits, or (c) the building of civic virtue and positive institutions” (Meyers et al. 2013, p. 620). We agree with Meyers et al. (2013, p. 618), who argue that such interventions “seem to be a promising tool for enhancing employee well-being and performance.”

### Limitations and Future Research

Our study is subject to some limitations that open up promising avenues for further research. First, current research is not free of skepticism about the concept of organizational psychological capital as the four components of psychological capital are interrelated (Dawkins et al. 2013). As it is the case with aggregated measures, future research might hence investigate the individual components of organizational psychological capital in addition to the composite score. Organizational psychological capital is also related to other concepts such as organizations’ emotional capability or regulatory focus. While the former relates to organizations’ acknowledging employee emotions and feelings (Huy 1999), the latter assesses whether organizations are motivated by promotion or prevention goals (Higgins and Pinelli 2020). Future research might strive to consider the relationships among these concepts.

Second, using computer-aided text analysis based on letters to shareholders, although well established in management research, entails some drawbacks. One may, for example, argue that letters to shareholders might reflect positivity not at the organizational, but at the management level; this, however, would still allow us to deduce valuable insights from our findings. Some scholars maintain that letters to shareholders might be subject to impression management intentions and thus reflect a rhetorical strategy of top executives rather than the actual shared level of positivity in an organization. Empirical work has countered this argument by validating a significant relationship between the content of letters to shareholders and firm outcomes (McKenny et al. 2013; Yadav et al. 2007), but future research would nevertheless benefit from alternative data generation approaches. Scholars seeking to capture information on a firm’s organizational psychological capital from more than one data source could, for example, expand content analyses to texts other than letters to shareholders. McKenny et al. (2018) point out, for example, that management discussion and analysis (MD&A) sections in 10‑K filings might represent an alternative inasmuch as they are not attributed to a specific group of employees and are usually more extensive than letters to shareholders. Scholars could also complement content-analytic measures of organizational psychological capital with survey results from members at multiple levels of an organization.

Third, the assessment of specific factor variance revealed that some words included in the dictionary of McKenny et al. (2013) were occasionally used out of context, even though the correlation between our manually coded scores and the computer-aided text analysis scores of organizational psychological capital exceeded 80%. Recognizing that participants sometimes used, for example, the word ‘energy’ in contexts in which it did not indicate *organizational hope* (e.g., energy drilling, energy sources, alternative energy), we follow McKenny et al.’s (2018) recommendation to refine the existing dictionary by iteratively updating the computer-aided text analysis measure, and by removing some words and adding others that might have been missing, but appear to be associated with a dimension of organizational psychological capital.

Fourth, our study is limited to large public U.S.-based family corporations listed in the S&P 500. Although top-tier family research has widely drawn on comparative samples (Anderson and Reeb 2003; Berrone et al. 2010; Dyer and Whetten 2006), such samples do not represent the full spectrum of family firms in the global economy. Small to medium-sized private family firms and family firms in different countries may behave differently from the U.S. firms in our sample. Previous studies show that governance approaches and founder families’ social embeddedness in firms substantially shape organizational structures, processes, and behaviors (Miller and Le Breton-Miller 2014; Miller et al. 2011). Replicating our study with a sample of family firms in a different regional context would provide an important contribution to existing theorizations of the heterogeneity of family firms. Additionally, observational secondary data offer limited potential to arrive at causal inferences; future work might incorporate research designs that allow causal patterns such as two-sided matching models (Chen et al. 2020). Our sample period ends in 2013—but particularly subdimensions of organizational psychological capital (i.e., resilience and optimism) have recently seen a surge in public attention due to the impact of the COVID-19 pandemic on firms. Future research could thus analyze later timeframes.

Last, we find that strong family influence exerted through TMT positions negatively relates to organizational psychological capital. This makes it increasingly important to identify means of enhancing organizational psychological capital without changing the composition of leadership teams. Memili et al. (2014) theorize that exchanges between family firm leaders and members might foster organizational psychological capital; other factors rooted in organizational culture and structure may similarly affect its development. Klein et al. (2005), for example, highlight how important family values are in shaping organizational firm culture and in cultivating a commitment to it. Investigating the influence of family values on organizational psychological capital—despite the immense difficulty in gathering reliable data—would constitute a major contribution to the field of positive organizational behavior and family firm research.
